# Early response of anti-vascular endothelial growth factor (anti-VEGF) in diabetic macular edema (DME) management: microperimetry and optical coherence tomography (OCT) findings: a pilot study at national eye center of third world country

**DOI:** 10.1186/s12886-024-03744-8

**Published:** 2024-12-27

**Authors:** Grimaldi Ihsan, Ameliza Kwartika, Made Indra Widyanatha, Rova Virgana, Erwin Iskandar, Arief Sjamsulaksan Kartasasmita

**Affiliations:** 1 Vitreoretina Department National Eye Center Cicendo Eye Hospital, Bandung, Indonesia; 2 Department of Opthalmology, Universitas Padjadjaran, Bandung, Indonesia

**Keywords:** Diabetic macular edema, Retinal sensitivity, Microperimetry, Macular thickness, Optical coherence tomography (OCT), Anti-VEGF response

## Abstract

**Purpose:**

To evaluate early response of retinal sensitivity (RS) and retinal morphology in diabetic macular edema (DME) patients after intravitreal anti-vascular endothelial growth factor (anti-VEGF) treatment.

**Methods:**

Sixteen eyes of 12 DME patients were included in this study conducted prospectively. All eyes underwent functional and morphologic examination of the macular area using microperimetry and optical coherence tomography (OCT) before and after intravitreal anti-VEGF injection. To determine significant differences between the values, paired *t* test was used. A correlation between CMT and RS was made using Spearman’s test.

**Results:**

Patients were evaluated at baseline, one week and one month after injection. The central macular thickness (CMT) decreased significantly from 449.33 *±* 100.79 μm to 427.94 *±* 85.76 μm to 357.93 *±* 75.92 μm. The RS improved significantly from 7.94 *±* 6.43 dB to 11.09 *±* 7.42 dB at one week and to 14.22 *±* 7.66 dB at one month after treatment. The CMT was significant negatively correlated to RS (*r=-0.259*, * p = < 0.001)*, with decay of 0.025 dB for every 1 μm increase of CMT.

**Conclusions:**

Retinal thickening due to DME can be adequately quantified using OCT, while microperimetry can offer information about retinal sensitivity in the exact location. Therefore, microperimetry can be a useful tool in predicting the functional outcome and determining the efficacy of anti-VEGF treatment for DME patients.

## Introduction

Diabetic Macular Edema (DME) is the leading cause of irreversible blindness in people with diabetes mellitus (DM), especially of productive age in developing countries. It is one of the biggest eye problems in the world [1, 2]. DME can occur at any degree of diabetic retinopathy or as a manifestation. DME is a complex long-term condition with multifactorial pathogenesis, including dysfunction of retinal pigment epithelium (RPE) and Müller’s cell, damaged Blood Retinal Barrier (BRB), non-perfusion of retinal capillaries and neuronal damage. All of this has implications for the complexity of the development of macular edema [3].

In 2018, the International Council of Ophthalmology (ICO) changed the classification of DME based on Optical Coherence Tomography (OCT) in DME management. ICO classifies DME into 3 degrees: without DME, non-center-involving DME (non-CI DME), and center-involving DME (CI-DME). Various parameters specific to the OCT picture have been proposed to evaluate DME and determine its various phenotypes, but the ICO classification is the most frequently used in everyday practice [2].

Vascular endothelial growth factor (VEGF) has implications for the pathogenesis of BRB changes and is believed to be a key mediator in the pathogenesis of DME. VEGF can trigger angiogenesis and cause BRB damage destructively, *as t*ight junction interstellar retinal endothelial cells play an essential role in the function and regulation of the BRB. This BRB damage then leads to the accumulation of plasma proteins such as albumin, causing an increase in the oncotic pressure of neural networks and resulting in interstitial oedema in the macula [2, 4]. Inflammation also plays a vital role in the process by involving several chemokines and cytokines. Many r*andomized controlled trials (RCTs)* show strong evidence supporting intravitreal anti-VEGF as a first-line therapy in managing sharp vision loss due to DME. Therefore, DME therapy’s goal has changed, namely from preventing blindness to improving vision. Anti-VEGF drugs such as bevacizumab, ranibizumab and aflibercept are very effective and safe, although bevacizumab is used regularly. It is more widely used in practice, is off-label, and is much cheaper [4, 5].

There is a lack of clear understanding of why DME causes visual impairment, and because visual function examination is limited to vision, there is little information in the literature about the effects of visual function corresponding to the specific location of lesions in diabetic retinopathy. In almost all RCTs, including Protocol V, the effectiveness of anti-VEGF therapy is evaluated based on visual functions such as *best corrected visual acuity (BCVA)* or anatomical parameters, such as retinal thickness determined by OCT examination. Most areas of the macula are involved in the reading process. At the same time, vision measurements with Snellen reflect only the function of one point in the central retina but do not provide information about the physiological status of the central retina [5, 6].

Microperimetry is a relatively new method, a subjective, quantitative, and non-invasive diagnostic test that can measure the physiological status of retinal sensitivity at different locations within 20 degrees of the center of the fovea and correlate it to the exact location of edema. Microperimetry is very useful in determining the locations that experience both relative and absolute scotoma and fixation characteristics. This parameter is very useful in calculating and predicting the effect of the function of macular edema or abnormalities in the macula [1, 5]. Microperimetry makes it possible to determine the sensitivity to increased light stimulus through each small area while looking at its projection on the fundus simultaneously at the right location [6, 7].

This study is first time conducted, aimed to assess the early retinal sensitivity and morphology response in DME patients given anti-VEGF therapy in the National Eye Center on the Third-World Country.

### Research methodology

This study is an analytical observational study of patients in the Vitreoretina unit of the National Eye Center Cicendo Eye Hospital Bandung during the research period from March to June 2022, prospectively. Sampling calculation was conducted to include all the patients that meet all criteria during period of study, thus using total sampling. All patients who were diagnosed with diabetic retinopathy with DME with fovea involvement *(center involved)* and who will receive intravitreal anti-VEGF therapy for the first time (treatment-naïve eye) were recruited in this study. All patients have undergone eye examinations including basic vision (uncorrected visual acuity = UCVA). This examination is conducted as this is the regulation on each department in the National Eye Center Cicendo Eye Hospital on daily basis to the patients. Other examinations include slit lamp, direct fundoscopy, non-contact tonometry, macular OCT and microperimetry before injection *of* the first anti-VEGF loading dose and repeated at one week post-injection and one month post-injection.

The degree of diabetic retinopathy (DR) is classified into five categories based on the International Clinical Diabetic Retinopathy Disease Severity Scale (DRSS), which are no DR, mild non-proliferative DR (NPDR), moderate NPDR, severe NPDR, and proliferative DR (PDR) [8]. Examination of each eye using Uncorrected Visual Acuity (UCVA) as measured by *logarithm of the minimum angle of resolution (LogMAR) chart.* Clinical diagnosis of DME based on clinical findings and obtained central macular thickness higher than baseline normal value on OCT machines. If there is OCT with poor reliability, significant cloudiness of refractive media, history of previous DME therapy (intravitreal or sub-tenon steroid injection and macular photocoagulation laser 3 months earlier), history of eye surgery three-months before, history of ocular or retinal abnormalities other than diabetic retinopathy, then excluded from this study.

### OCT examination

**Fig. 1:**
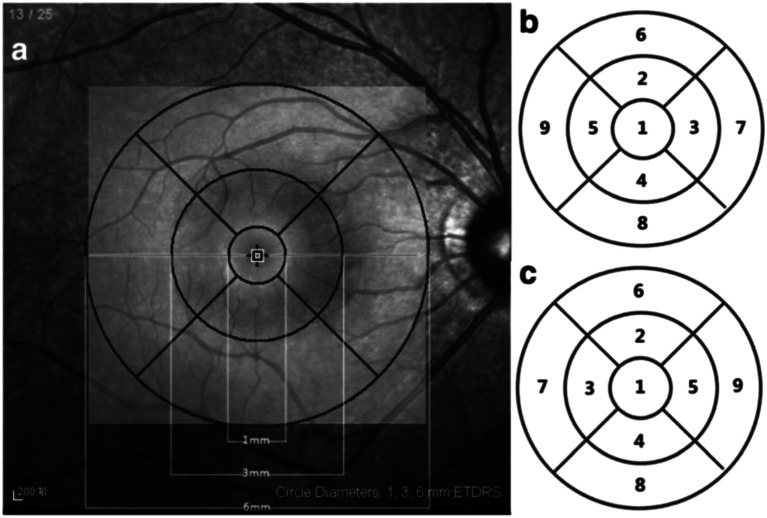
Standard layout of ETDRS grid. (**a**). Display of 9 macular subfile areas based on ETDRS. (**b**). 9 areas of the ETDRS subfield of the right eye. (**c**). 9 subfields of ETDRS of the left eye[10] Source: 10.1186/s12886-018-0842-y.

OCT performed using a Stratus OCT scanner (Cirrus HD 5000; Carl Zeiss Meditech GmbH, Germany). The scan protocol used in this study was the Fast Macular Thickness program, which produced retinal mapping consisting of 6 radiating cross-sectional scans, each 6 mm length that produced a circular plot where the fovea become a central zone of 1mm diameter. The superior, nasal, inferior, and temporal parafoveal zones are shown as rings in these areas. There are two another concentric zones which are 3mm and 6mm. The nine OCT zones are referred to as ETDRS-type regions because they are like the zones in the ETDRS fundus photo analysis (Fig. 1). Macular thickness is described by color values, where brighter colors indicate an increase in retinal thickness, which is measured automatically and compared to normative data contained in OCT according to age groups [1, 9].

### Microperimetry

Automatic Fundus-Related Perimeter MP-3 (Nidek Technologies, Gamagori, Japan) were used as microperimetry, and is the latest generation microperimetry, with a stimulus of 20 degrees from the central fovea and a wide dynamic range between 0 and 34 dB with a maximum luminance of 10,000 apostilbs (asb). The fundus image is displayed in high resolution by the fundus scanning laser ophthalmoscope (SLO) photo on the video screen in real time, so that the operator can see how it looks on the retina on the screen. Target fixation and stimulus are projected automatically onto the retina quickly and precisely with eye tracking technology that compensates for eye movements. This makes the expected position of the stimulus fall precisely and specifically at the intended location of the retina. This function provides convenience during follow-up [1, 11].

All patients in this study were examined microperimetry in dilated pupils, using the following parameters: There were 33 stimuli mapped to 12^0^ from the central fovea, the fixation target consists of a red ring with a diameter of 1^0^, against the background of white monochromatic luminance at 31.4 asb; stimulus size using Goldman III (white, projected duration 200 ms); and Threshold Strategy 4 − 2 [1, 11]. The grid stimulus consists of a single response from the central fovea and 4 concentric rings at the retinal locus   1^0 ^, 2^0^, 4^0^, and 6^0^ from the central point. Normal values of retinal sensitivity in MP-3 are above 27 dB shown in green. Fixation stability is grouped as stable, relatively unstable, and unstable using Fuji’s Classification this microperimetric examination is considered reliable when the response *false positives* still below 25% [1, 7, 12].

To accurately correlate retinal thickness data on OCT with retinal sensitivity data on microperimetry, this study used only subfield areas 1–5 and ignored subfield areas 6–9 based on the ETDRS grid on OCT (Fig. 1).

### Anti-VEGF

The anti-VEGF agent used in this study was bevacizumab (Bevaas, Hetero Biopharma Limited, India). Patients under local anesthesia, aseptic action is performed, followed by intravitreal anti-VEGF injection of 0.05 ml (1.25 mg) with a sterile 30-gauge needle [5]. All patients are planned to receive injections at least 3 times 1 month apart. Post-injection all patients were given topical antibiotic eye drops (ofloxacin 0.3%).

### Statistical analysis

Almost all variable are numerical, thus the data showed by mean, standard deviation, median, and range (minimal-maximal value). On the other hand, categorical data presented using proportion and percentage. Correlation between CMT and RS calculated using spearman correlation coefficient. Linear regression analysis also conducted in which CMT refer as indepentend variable and RS as dependent variable. Analysis for RS each RS and CMT in different times (before injection, one week after injection, and one month after injection) used paired t test analysis by comparing two values in different time. Statistically significant analysis show by *p* value < 0.05. All analysis conducted using SPSS ver. 26

**Fig. 2:**
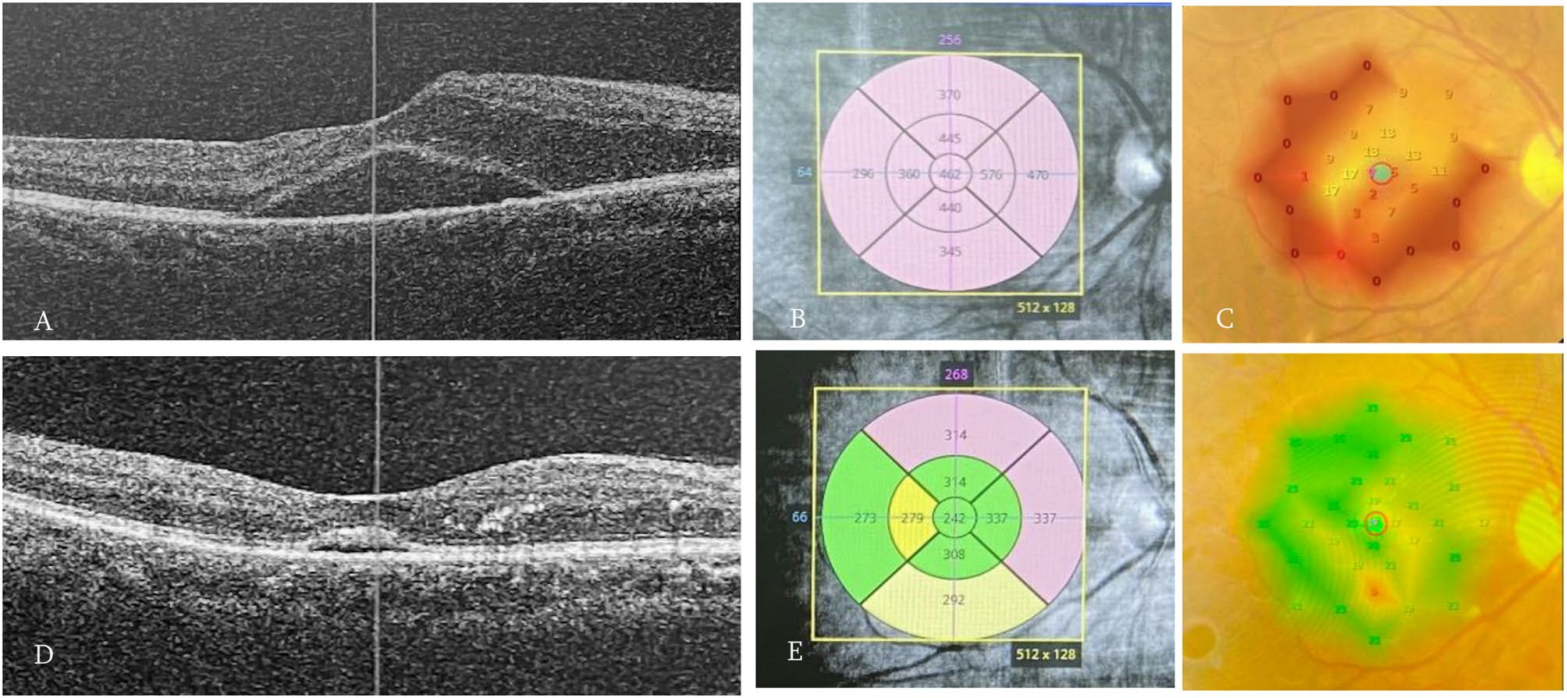
Microperimetry (in dB) is superimposed over a color fundal photo in the case of DME with hard exudate. Decreased retinal sensitivity is especially noticeable in the central and inferior areas of the macula. (**A**) The visit prior to the injection of anti-VEGF, UCVA 0.8 LogMAR, showed fluid in the intraretinal layer, accompanied by accumulation of subretinal fluid causing *serous foveal detachment*. (**B**) *OCT map*, CMT in area 1 is 402 μm. (**C**) *RS threshold* in MP-3 in corresponding area is 7 dB. (**D**) 1 month after the injection of the first *loading dose* of bevacizumab, the resolution of DME appears. (**E**) CMT in area 1 drops to 242 μm. (**F**) and *RS threshold* at exactly the same retinal location as before injection improves to 17 dB. The same is true in areas 2–5 of ETDRS

## Result

Table 1 shows that there were 16 eyes from 12 patients with center involved *DME* (5 men and 7 women), with an age range of 52–69 years (mean 57.25 ± 4.29 years) included i*n* this study. All patients were patients with type 2 DM, and 66.67% of them were with bilateral CI-DME. From all 16 eyes involved in the research, there are 11 eyes with severe NPDR and 5 eyes with PDR. The duration of suffering from DM ranges from 2 to 21 years (average 8 ± 5.74), while blurred eye complaints on average have only been felt for 46.67 days. The visual acuity when the patient comes is around 1.04 + 0.42 LogMAR.

The average hemoglobin A1c content (HbA1c) (%) is 8.51 ± 2.08 (range, 6–12). From 12 patients There were 75% of patients with hypertension and 83.3% with dyslipidemia. The mean baseline intraocular pressure is 15.08 + 3.52 mmHg. Among the 16 eyes there were 2 eyes with a history of cataract surgery and 2 eyes with a history of previous PRP laser. Patients with history of PRP is only in one site no in multisite position. The stability of fixation at the time of microperimetry examination mostly indicates a stable condition both before and after anti-VEGF injection.

**Table 1:** Patients characteristics

Variable	Sum	%
Number of patients	12	
Number of Eyes	16	
Age (years)		
Mean ± SD	57.25 *±* 4.29	
Median (Range)	56.5 (52–69)	
Gender		
Male: Female	5: 7	41,67: 58,33
DM Type		
Type 1: Type 2	0: 12	0: 100
DM duration (years)		
Mean ± SD	8 ± 5.74	
Median (Range)	7 (2–21)	
Blurred vision duration (days)		
Mean ± SD	46.67 ± 32.50	
Median (Range)	30 (15–120)	
Hypertension	9	75
Dyslipidemia	10	83,3
UCVA-baseline, logMAR		
Mean ± SD	1.04 ± 0.42	
Median (Range)	1.05 (0.5–1.8)	
HbA1c levels		
* <6.4%*	2	16,67
>6.5%	10	83,33
IOP		
Mean ± SD	15.08 ± 3.52	
Median (Range)	14 (8–21.40)	
CI-DME laterality		
Unilateral: Bilateral	4: 8	33,33: 66,67
Degrees of Diabetic Retinopathy		
Severe NPDR: PDR	11: 5	68,75: 31,25
Previous actions		
Laser PRP	2	12,5
Cataract surgery	2	12,5
Fixation stability		
Pre injection		
-Stable	10	62,5
- Relatively unstable	6	37,5
- Unstable	-	-
Post-injection 1 month		
-Stable	11	68,75
- Relatively unstable	5	31,25
- Unstable	-	-

UCVA = uncorrected visual acuity, HbA1c = haemoglobin A1c, TIO = intraocular pressure, CI-DME = center involving-DME, PRP = pan retinal photocoagulation

**Fig. 3:**
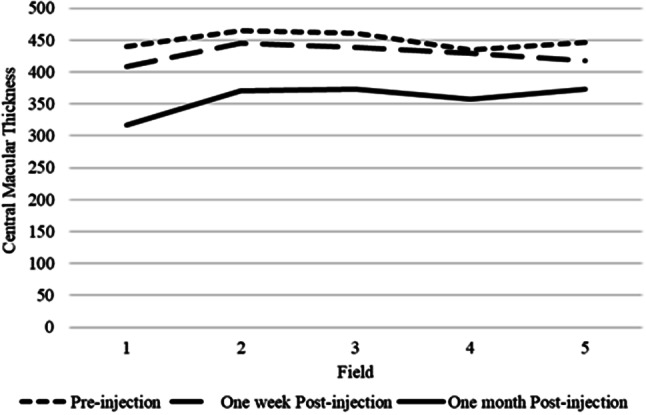
Macular thickness based on examination area (1. central, 2. superior, 3. temporal, 4. inferior, 5. nasal) and time of anti-VEGF injection

**Fig. 4:**
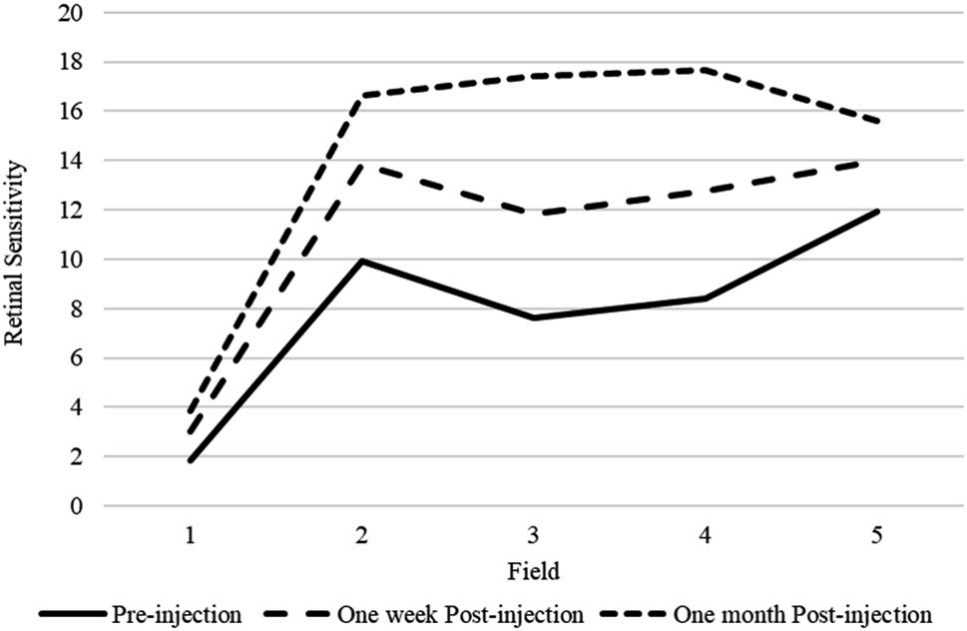
Retinal sensitivity based on examination area (1. central, 2. arrogant, 3. temporal, 4. inferior, 5. nasal) and antiVEGF injection time

Based on Fig. 2 and 3, macular thickness is shown based on the examination area. In this graph it appears that before the anti-VEGF injection, the area with the highest thickness was in area 2 of 465.25 μm and the lowest in area 4 of 434.31 μm. Post 1-week injection, the highest CMT appears to be in area 2 at 445.25 μm and the lowest at area 1 at 408.63 μm. After 1 month post injection, the highest CMT was in area 5 at 373.63 μm and the lowest at area 1 at 316.25 μm. Overall, it appears that post-1-month injection CMT has a lower value than post-injection 1-week, as well as post-injection 1-week appears lower than before injection.

Figure 4 shows retinal sensitivity (RS) based on the examination area. Before the anti-VEGF injection, it appeared that the lowest RS was in area 1 which was 1.83 dB while the highest in area 5 was 11.91 dB. One week after injection, it appears that the lowest RS is in area 1 which is 3.02 dB and the highest in area 5 which is 13.99 dB. After 1 month post injection, it appears that the lowest RS is in area 1 which is 3.83 dB while the highest in area 4 is 17.66 dB. Overall, it appears post-injection RS 1 months higher than post injection 1 week, as well as RS post injection 1 week appears higher than before injection.

The relationship between CMT and RS analysed using the *Spearman Rank Correlation* test at a significant level of 5% (Table 2).

**Table 2:** CMT and RS correlation test results

Variable	*R*	*p*-value
Macular thickness & retinal sensitivity	-0,259	< 0.001*

*Significantly related if *p* < 0.05

**Fig. 5:**
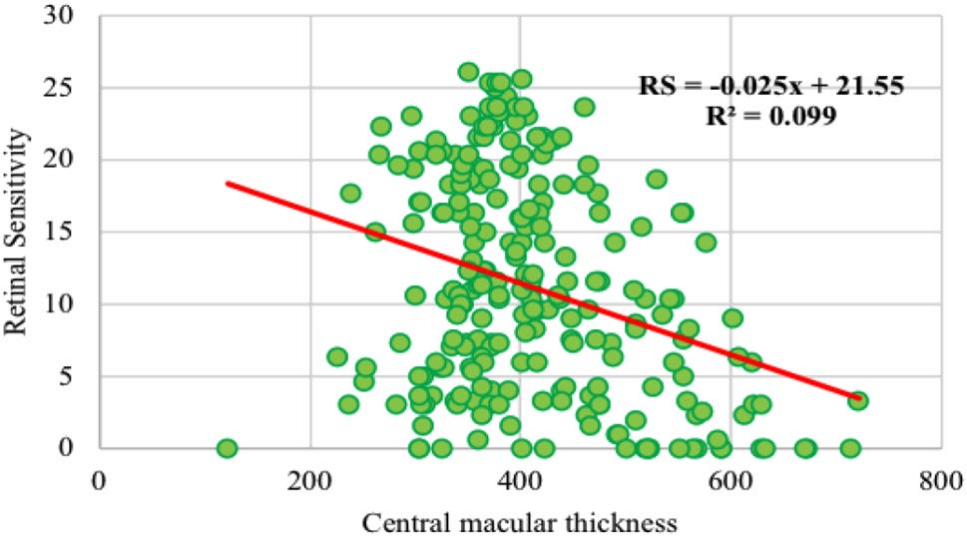
Linear regression of central macular thickness on retinal sensitivity

Table 2 show a results of the CMT correlation test with RS have a value of p = < 0.001 smaller than 0.05, so it is concluded that there is a significant negative correlation *r* = -0.259 between CMT and RS. The degree of relationship between them is moderate and negative because the value of r is close to 0.5 and is negative. Due to the significant correlation relationship, it proceeded to linear regression analysis to see the effect of CMT on RS Fig. 5.

In linear regression analysis on the Fig. 5, the independent variable is CMT and the dependent variable is RS. Based on the results of the t test obtained *p = 0.000 < 0.05 and* and t=-5.226 > -t (0.025l238) = 1.97 then it can be concluded that there is a relationship between the negative influence of CMT on RS. The values of the coefficients in the retinal sensitivity regression model are:

Y = 21.550–0.025 (X1)

The retinal sensitivity regression model means that if the CMT value increases by 1 μm then the RS will decrease by 0.025 dB. If the increase in CMT is absent or 0 then the RS is 21,550 dB. The value of a*djusted R square* is obtained at 0.099 which means that the CMT variable can explain RS by 9.9% and the other 90.1% is explained by variables other than CMT.

Differences in retinal sensitivity before and after anti-VEGF injection in this study will be analyzed using *paired sample t-test.* Table 3 shows that the RS hospital at the time of pre-VEGF injection was 7.94 ± 6.43 dB, 1 week after injection retinal sensitivity increased by 3.15 dB to 11.09 ± 7.42dB. The results of statistical analysis paired *sample t-test* have a value of *p* < 0.001, which means that there is a significant difference in the patient’s retinal sensitivity before and 1 week post injection of anti-VEGF where after 1 week post injection there is an improvement in retinal sensitivity compared to before injection.

**Table 3:** Differences in retinal sensitivity before and after anti-VEGF injection

Variable	Treatment	Mean ± SD (dB)	Difference	*p*-value
Retinal Sensitivity (RS)	Pre Injection	7.94 *±* 6.43	-3.15 *±* 5.12	< 0.001*
	Post 1 Week	11.09 *±* 7.42		
	Pre Injection	7.94 *±* 6.43	-6.29 *±* 6.61	< 0.001*
	Post 1 Month	14.22 *±* 7.66		
	Post 1 Week	11.09 *±* 7.42	-3.14 *±* 5.18	< 0.001*
	Post 1 Month	14.22 *±* 7.66		

*Significantly related if *p* < 0.05

After 1 month post injection, retinal sensitivity also increased by 6.29 dB to 14.22 *+ 7.66* dB. The results of statistical analysis of *paired sample t-test* have a value of *p* < 0.001, which means that there is a significant difference in the patient’s retinal sensitivity before and 1 month after injection where there is an improvement in retinal sensitivity after injection.

**Fig. 6:**
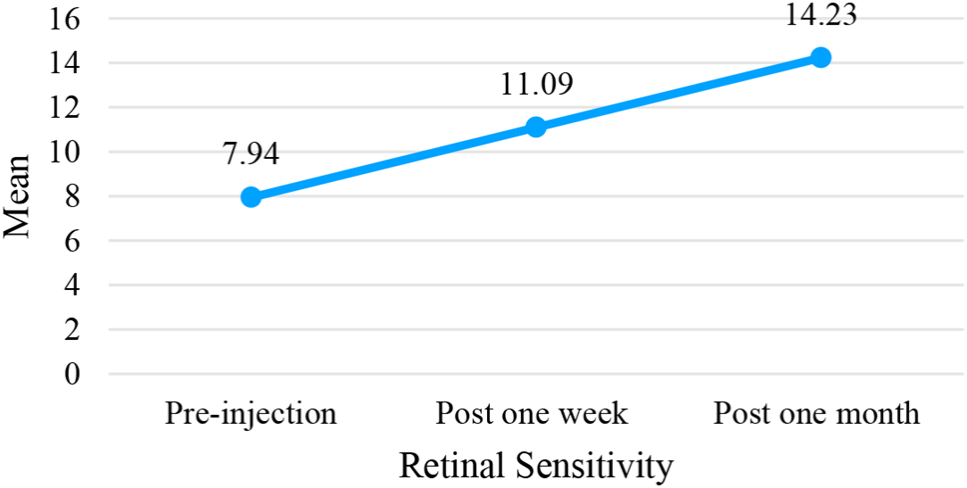
Retinal sensitivity before injection, post-injection one week, and post-injection one month

The difference in RS after 1 week and 1 month post-injection has a significantly different value with *p < 0.001* smaller than the significant level of 0.05. This means that after 1 month of antiVEGF injection, the average RS is 14.22 *±* 7.66 dB, which gives a much greater increase than after 1 week, which is 11.09 *±* 7.42 dB. The illustration of the results of increased retinal sensitivity since pre-injection, 1 week to 1 month post-injection can be described in Fig. 6.

Figure 6 shows that the average RS before injection was 7.94 dB, then one week after injection there was an increase to 11.09 dB, as well as one month after injection it appeared that the patient’s retinal sensitivity increased to 14.23 dB. This means that administering anti-VEGF to patients can improve retinal sensitivity.

Table 4 shows the mean CMT in patients before anti-VEGF injection was 449.33 *+ 100.79* μm, and 1 week after injection there was a decrease of 21.39 μm to 427.94 *+ 85.76* μm. The results of statistical analysis paired *sample t-test* have a value of *p* < 0.001, which means that there is a significant difference in the patient’s CMT before injection and 1 week post injection. This means that 1 week after the injection there is a decrease in macular thickness compared to before the anti-VEGF injection.

**Table 4:** Differences in central macular thickness before and after anti-VEGF injection

Variable	Treatment	Mean ± SD (µm)	Difference	*p*-value
Central macular thickness (CMT)	Pre Injection	449.33 *±* 100.79	21.39 *±* 47.72	< 0.001*
	Post 1 Week	427.94 *±* 85.76		
	Pre Injection	449.33 *±* 100.79	91.40 *±* 98.38	< 0.001*
	Post 1 Month	357.93 *±* 75.91		
	Post 1 Week	427.94 *±* 85.76	70.01 *±* 77.47	< 0.001*
	Post 1 Month	357.93 *±* 75.91		

*Significantly related if *p* < 0.05

At 1 month post anti-VEGF injection, the CMT decrease of 91.40 μm to 357.93 ± 75.91 μm. The results of the paired *sample t-test statistical analysis* have a value of *p* < 0.001,* meaning that* there is a significant difference in the patient’s CMT before and 1-month post-injection. This means that after 1-month post-injection there is a decrease in macular thickness compared to before anti-VEGF injection.

**Fig. 7:**
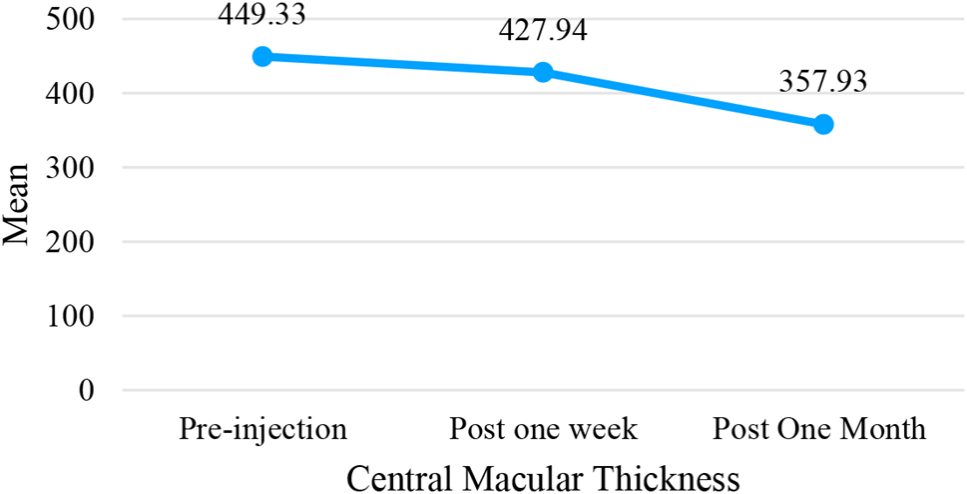
Macular thickness pre injection, post one week, post one month

The difference in macular thickness in patients during 1-week injection and 1-month injection has a significantly different value with *p < 0.001.* This means that after 1-month post-injection the average CMT is 357.93 ± 75.92 μm, which means there is a much greater decrease than 1 week after injection. Figure 7 shows a illustration of the results of CMT decline since before injection, 1 week to 1 month.

In Fig. 7, it can be seen that the average CMT before injection was 449.33 μm, and 1 week post-injection there was a decrease to 427.94 μm, and after 1 month CMT seemed to decrease to 357.93 μm. This means that administering anti-VEGF to DME patients can decrease the thickness of the central macula.

## Discussion

Macular edema represents the primary etiology of visual acuity deterioration within the diabetic population. Established risk factors for DME development include the duration of diabetes mellitus (DM), HbA1c levels, and blood pressure. Several studies have demonstrated that these factors are key determinants in diabetic retinopathy (DR) pathogenesis. However, even with good glycemic control, halting the progression of DR is not guaranteed [2, 13].

In this study, the diagnosis of type 2 DM, hypertension and dyslipidemia was established by an internist whose results were obtained from electronic medical record data. DME is more common in patients with type 2 diabetes than type 1, as well as the duration of suffering from DM. Hyperglycemia leads to the accumulation of Advanced Glycation End Product and oxygen free radicals, which cause the activation of inflammatory pathways. Therefore, the longer a person suffers from DM, the longer the retina is exposed to hyperglycemia, then will induce further damage. Several studies have shown that hyperglycemia and high levels of HbA1c are closely related to the incidence and progression of DR at follow-up over 4 years in type 2 DM, including the degree of PDR [2] Other studies emphasize that tighter, earlier control of glycemia will be effective in preventing or slowing the onset of diabetic retinopathy and reducing the risk of DME [13].

Hypertension is another established comorbidity in DME pathogenesis, potentially exacerbating retinal vascular damage through hyperperfusion and elevating the risk of tissue edema. Some studies show that with good blood pressure control will reduce the risk of diabetic retinopathy by 34% [13]. In this study, most patients had comorbid hypertension and dyslipidemia as well as high HbA1c levels, coupled with a long duration of DM. The PRP effect on retinal sensitivity showed when the approach for the procedure was multispot in patient with diabetic retinopathy [14]. Our patients did not get this approach of multispot, thus it didn’t affect the result for microperimetry measurements.

Ocular risk factors including a history of previous cataract surgery are said to be associated with the progression of diabetic retinopathy and the occurrence of DME. Although DME can occur without diabetic retinopathy, DME is closely related to the severity of diabetic retinopathy [13] The precise duration of DME remains elusive; however, it is often estimated based on the initial presentation of visual decline. The longer occurrence of DME will have an impact on the resistance and function of macular cells that experience mechanical and toxic stress due to edema. In patients with DME, photoreceptor damage appears to occur as a final phenomenon, and may not be related to the formation of intraretinal cysts, but rather to the preceding process of retinal neurodegeneration [1, 15].

Diagnosis of DME is achieved through biomicroscopic fundus examination, fundus photography, and OCT. OCT has emerged as the gold standard for quantifying and monitoring macular edema, including DME. In a study conducted by Vujosevic et al. it was stated that a decrease in retinal sensitivity is related to an increase in retinal thickness [1]. In a large population study involving 357 subjects with DM, there was a decrease in mean retinal sensitivity that was significantly associated with central fovea thickness in subjects without DR. This suggests that in patients with DR there is earlier neuronal degeneration damage before the onset of DR [16] Another study, which examined more about the structure-function relationship of the retina, showed a meaningful relationship between retinal sensitivity and ganglion-inner retinal cell layer thickness (GCL-IPL) in subjects with DM, but not in healthy people as controls. The result showed there was no significant difference in BCVA between the two groups, indicating that neuronal damage may occur in individuals whose vision may be normal [17]. Another study reported an association between retinal sensitivity measured by microperimetry and vision in patients with DME. Although vision is an important parameter for assessing physiological function, it does not always reflect visual function comprehensively because it only measures retinal function in 1^0^central field of view [15].

Microperimetry seems to display better retinal function than BCVA in calculating visual function in DM patients [1] Retinal sensitivity as measured by microperimetry has been used as an important measure of retinal function in DME evaluation. There is an association between retinal sensitivity and macular thickness in patients with CSME, where there is a loss of 0.83 dB for every 10% deviation from normal retinal thickness [1, 12] In this study, microperimetric examination showed that macular sensitivity decreased significantly in DME, where the more the thickens retina, the more the retinal sensitivity decreased. From the results of linear regression analysis, it was found that every increase of 1 μm of retinal thickness, there will be a decay of retinal sensitivity by 0.025 dB.

Retinal sensitivity at 1 week after intravitreal bevacizumab injection began to show improvement. This is thought to be related to the pharmacokinetics of the anti-VEGF agent. From studies conducted by Synapse et al. in rabbits, it was said that the half-life of bevacizumab in vitreous was 6.61 days, the highest concentration of bevacizumab in vitreous occurred on the first day after injection and then decreased at week 4, but remained at a high level even up to 29 days post-injection. Zhu et al. reported the maximum concentration of bevacizumab in the patient’s vitreous occurred on day 2 after injection of 1.25 mg of intravitreal bevacizumab [18] Bakri et al. reported that the half-life of bevacizumab in vitreous in rabbits was 4.32 days, longer than ranibizumab which was only 2.88 days. This is due to the greater molecular weight of bevacizumab (149 kDa) than ranibizumab (48 kDa). Therefore, the penetration of bevacizumab into the retina and *its clearance* from the vitreous becomes slower so that its half-life becomes longer [19, 20].

Research in microperimetry examination after intravitreal injection conducted by Malagola et al. showed improvement in 76.9% eyes after repeated administration in 4-week interval for three consecutive time [21] Our findings are giving the information examination in early stage at one day, one week, and lastly 4 weeks after injection, thus the early findings are stressing out. Both show a significant difference, giving an input where earlier examination can also be done. Gonzales et al. on the other hand using different drugs for intravitreal injection, which is aflibercept. The findings showed that an improvement of BCVA and retinal sensitivity [22] On the other hand, our findings using UCVA approach as our institution policy, which giving another point of view.

In DME, microperimetry has been used to measure macular sensitivity, the relationship between macular sensitivity to macular thickness and visual acuity, also determine fixation characteristics at various degrees and types of DME [15]. Fixation characteristics (location and stability) are parameters related to the quality of the patient’s vision, especially the ability to read. The ability to read is said to have more to do with the quality of subjective vision than far vision [23, 24] Vujosevic et al. report that the characteristic pattern of fixation is not significantly affected either topographically by edema expansion (focal or diffuse) or on the basis of DME classification with OCT [23]. The stability of fixation in the eye with DME shows that in eyes with DR photoreceptor damage is not the main thing, but rather vascular and neuronal damage [25]. Another study states the only parameter affecting fixation is the presence of hard exudate sub-fovea [15].

The stability fixation shown in MP-3 results used the Fuji’s Classification which was calculated based on the percentage of fall of the patient’s fixation point within radius 2^0^ and 4^0^ from the fixation target center *(Red Circle)*. Fixation is said to be stable when the fixation points of > 75% falls at 2^0^*Circle*, relatively unstable when < 75% falls at 2^0^*Circle* or > 75% falls at 4^0^*Circle* and is said to be unstable when < 75% falls at 4^0^*Circle.* In this study, there was almost no change in fixation patterns either before or after anti-VEGF therapy, with most fixation patterns stable.

Microperimetry may be valuable in estimating the final function of DME after intervention and seems to be in line with the return of fovea thickness as also seen in the results of this study. The hypothesis has also been substantiated by a prospective randomized study conducted by Vujosevic et al. [1, 15]. However, several limitations were reported as it only covered patients that were suitable for the criteria and only included a limited number of patients. Another thing is, as the study only evaluates the response up to the first month after the first dose of anti-VEGF, making short observations. Lastly, only one type of anti-VEGF agent is evaluated so it cannot give comparation to other anti-VEGF that are commonly used.

## Conclusion

The results of this study suggest that microperimetry can be utilized in determining the efficacy of anti-VEGF therapy in DME patients. Supporting modalities such as OCT and microperimetry can provide precise information about the correlation between macular structure-function before and after therapy. And most importantly, this is expected to help increase patient adherence and confidence in the results of therapy.

## Data Availability

The data that support the findings of this study are owned by and exclusively stored at National Eye Center, Cicendo EYE Hospital, Indonesia. Requests for access to the data can be directed to Research Division or the corresponding author, who can facilitate the data access process.
